# Identification of important modules and biomarkers in tuberculosis based on WGCNA

**DOI:** 10.3389/fmicb.2024.1354190

**Published:** 2024-02-08

**Authors:** Jing Dong, Ruixue Song, Xuetian Shang, Yingchao Wang, Qiuyue Liu, Zhiguo Zhang, Hongyan Jia, Mailing Huang, Chuanzhi Zhu, Qi Sun, Boping Du, Aiying Xing, Zihui Li, Lanyue Zhang, Liping Pan, Zongde Zhang

**Affiliations:** ^1^Beijing Chest Hospital, Capital Medical University, Beijing, China; ^2^Beijing Key Laboratory for Drug Resistant Tuberculosis Research, Beijing, China; ^3^Beijing Tuberculosis and Thoracic Tumor Research Institute, Beijing, China; ^4^Department of Intensive Care Unit, Beijing Chest Hospital, Capital Medical University, Beijing, China; ^5^Changping Tuberculosis Prevent and Control Institute of Beijing, Beijing, China; ^6^Department of Tuberculosis, Beijing Chest Hospital, Capital Medical University, Beijing, China

**Keywords:** tuberculosis, lncRNA, mRNA, WGCNA, diagnosis

## Abstract

**Background:**

Tuberculosis (TB) is a significant public health concern, particularly in China. Long noncoding RNAs (lncRNAs) can provide abundant pathological information regarding etiology and could include candidate biomarkers for diagnosis of TB. However, data regarding lncRNA expression profiles and specific lncRNAs associated with TB are limited.

**Methods:**

We performed ceRNA-microarray analysis to determine the expression profile of lncRNAs in peripheral blood mononuclear cells (PBMCs). Weighted gene co-expression network analysis (WGCNA) was then conducted to identify the critical module and genes associated with TB. Other bioinformatics analyses, including Kyoto Encyclopedia of Genes and Genomes (KEGG), Gene Ontology (GO), and co-expression networks, were conducted to explore the function of the critical module. Finally, real-time quantitative polymerase chain reaction (qPCR) was used to validate the candidate biomarkers, and receiver operating characteristic analysis was used to assess the diagnostic performance of the candidate biomarkers.

**Results:**

Based on 8 TB patients and 9 healthy controls (HCs), a total of 1,372 differentially expressed lncRNAs were identified, including 738 upregulated lncRNAs and 634 downregulated lncRNAs. Among all lncRNAs and mRNAs in the microarray, the top 25% lncRNAs (3729) and top 25% mRNAs (2824), which exhibited higher median expression values, were incorporated into the WGCNA. The analysis generated 16 co-expression modules, among which the blue module was highly correlated with TB. GO and KEGG analyses showed that the blue module was significantly enriched in infection and immunity. Subsequently, considering module membershi*p* values (>0.85), gene significance values (>0.90) and fold-change value (>2 or < 0.5) as selection criteria, the top 10 upregulated lncRNAs and top 10 downregulated lncRNAs in the blue module were considered as potential biomarkers. The candidates were then validated in an independent validation sample set (31 TB patients and 32 HCs). The expression levels of 8 candidates differed significantly between TB patients and HCs. The lncRNAs ABHD17B (area under the curve [AUC] = 1.000) and ENST00000607464.1 (AUC = 1.000) were the best lncRNAs in distinguishing TB patients from HCs.

**Conclusion:**

This study characterized the lncRNA profiles of TB patients and identified a significant module associated with TB as well as novel potential biomarkers for TB diagnosis.

## 1 Introduction

Tuberculosis (TB), which is caused by infection with *Mycobacterium tuberculosis* (*M.tb*), is an epidemic disease of global health concern. Approximately one-fourth of the global population is estimated to have been infected with *M.tb*, but only a small number of people develop active tuberculosis (ATB) each year (Bagcchi, 2023). Nevertheless, TB remains a leading cause of death worldwide. Although numerous mechanistic studies have examined *M.tb* infection and TB development in recent years, the role and mechanism of important molecules remain largely unexplored (Fathizadeh et al., 2020). Obtaining a better understanding of the underlying pathogenesis and regulatory network may facilitate the development of methods to prevent or control TB.

Recently, the development of high-throughput genome-wide gene analysis technologies, such as microarray, next-generation sequencing, and single-cell transcriptome and novel microarray-based integrated bioinformatics analyses, have helped promote the screening and identification of pivotal biomarkers associated with diseases and further elucidate the mechanisms underlying TB occurrence and development (Li et al., 2022; Salmen et al., 2022; Zhu et al., 2023). Until now, the Xpert MTB Host response assay [including Dual specificity phosphatase 3 (DUSP3), Guanylate-binding protein (GBP5), Krupple-like factor 2 (KLF2) genes] which was developed by Cepheid (Sunnyvale, CA, United States) has been recommended in TB screening by WHO (Sodersten et al., 2021; Wu et al., 2023). Other transcriptomic signatures, such as RISK6 Host response assay (QuantuMDx, United Kingdom), IRISA-TB (Antrum Biotech, South Africa), T cell activation marker (TAM-TB) assay (Ludwig-Maximilians-University, Germany), and so on, were also in development (Penn-Nicholson et al., 2020). Therefore, there is no doubt that transcriptomic signatures based on host immune response to *M.tb* or other mechanisms have the potential in diagnosis of TB, and WHO has also recommended to develop the host biomarker-based assay for TB diagnosis (Penn-Nicholson et al., 2020). In the human genome, most nucleic acids are noncoding RNAs (ncRNA), which are thought to play important roles in various biological processes. Furthermore, based on the developed technologies, it is possible to quantify the specific ncRNA molecules in cellular and subcellular compartments of diseased cells, as well as in extracellular compartments (such as exosomes and body fluids), which makes these molecules suitable for liquid biopsy utility (Nemeth et al., 2023). Approximately three-fourths of ncRNAs are long noncoding RNAs (lncRNAs), which have a length of over 200 nucleotides and tissue/cell-specific expression patterns. Previous research has suggested that lncRNAs are involved in regulating gene expression via interactions with common biological macromolecules, forming a complex network that regulates multiple normal biological and disease processes (Fathizadeh et al., 2020; Liang et al., 2022). A number of studies have examined the expression and function of lncRNAs in various diseases based on co-expression analyses (Liu et al., 2022; Wen et al., 2022). Although there is no commercial assay based on lncRNAs in TB diagnosis, many researches have confirmed that the abnormal expression of lncRNAs are associated with TB occurrence, development and prognosis, and have the potentials as diagnostic, prognostic biomarkers and therapeutic targets in TB (Chen et al., 2017; Zhang et al., 2022; Xu et al., 2023). However, the expression patterns and pathogenesis of host lncRNAs in TB patients have not yet been fully elucidated, and mechanistic details regarding the regulatory network involving lncRNAs in TB remain unclear (Chen et al., 2017; Agliano et al., 2019; Liang et al., 2022). Uncovering the expression profile and co-expression relationship between ncRNAs and mRNA in the host could facilitate the development of novel strategies for TB prevention and therapy (Xia et al., 2023).

In the present study, we performed a genome-wide ceRNA microarray analysis of peripheral blood mononuclear cells (PBMCs) from TB patients and health controls (HCs) to elucidate lncRNAs profile associated with TB. We also performed a weighted gene co-expression network analysis (WGCNA) to identify important expression modules associated with TB. The results of this study shed light on the gene expression profile in TB patients and provide new clues for exploring the regulatory mechanisms of lncRNAs in the pathogenesis of TB.

## 2 Materials and methods

### 2.1 Ethical approval

This study was performed in accordance with the guidelines of the Helsinki Declaration and was approved by the Ethics Committee of the Beijing Chest Hospital, Capital Medical University. Written informed consents were obtained from each participant before blood collection.

### 2.2 Participants information

TB patients in the discovery set were recruited from Beijing Chest Hospital between January 2019 and May 2019. HCs in the discovery set were enrolled from a TB screening campaign in Beijing Changping District between October 2019 and December 2019. TB patients in the validation set were recruited from Beijing Chest Hospital between December 2021 and August 2022, and HCs in the validation set were enrolled from a physical examination program conducted at Beijing Chest Hospital between October 2021 and December 2021. TB patients were diagnosed based on positive *M.tb* culture, positive Xpert MTB/RIF, positive microscopy, or positive histology. All enrolled HCs were confirmed as not infected with *M.tb* based on normal computed tomography results and negative T-SPOT.TB results (Kruse et al., 2021). Individuals positive for human immunodeficiency virus (HIV), hepatitis B virus (HBV), hepatitis C virus (HCV), diabetes, severe autoimmune diseases, or those who took immunosuppressive or immunopotentiator agents, received anti-TB treatment, or were pregnant or lactating were excluded.

### 2.3 Blood sample collection

Peripheral blood (3 mL) was collected from each individual into heparin-containing vacutainer tubes. PBMCs were isolated by density gradient using Lympholyte Cell Separation Media (HY2015, Tianjin Haoyang Biological Manufacture Co., Ltd., China) within 4 h after blood collection. The isolated PBMCs were lysed with TRIzol reagent (Invitrogen, Carlsbad, CA, United States) and stored at −80°C to avoid RNA degradation. The samples were not thawed repeatedly.

### 2.4 RNA extraction

Total RNA was extracted from PBMCs using a miRNeasy Mini kit (217004, QIAGEN, Germany) according to the protocols recommended by the manufacturer. RNase-free DNase I (79254, QIAGEN) was added to remove genomic or cell-free DNA contamination. The integrity and quality of RNA from PBMCs were evaluated using an Agilent 2,100 Bioanalyzer (Agilent Technology, Palo Alto, CA, United States). RNA with a 2,100 RNA integrity number ≥ 7.0 and 28S/18S > 0.7 was used for the microarray study and qPCR validation.

### 2.5 Microarray study

Each slide was hybridized with 1.65 μg of Cy3-labeled cRNA using a Gene Expression Hybridization kit (5188-5242, Agilent Technologies, Santa Clara, CA, United States) and hybridization oven (G2545A, Agilent Technologies) according to the manufacturer’s instructions. After 17 h of hybridization, slides were washed in staining dishes (121, Thermo Shandon, Waltham, MA, United States) using a Gene Expression Wash Buffer kit (5188-5327, Agilent Technologies) according to the manufacturer’s instructions. The slides were then scanned using an Agilent Microarray Scanner (G2565CA, Agilent Technologies) with the following default settings: dye channel, Green; scan resolution, 3 μm; PMT, 100%; 20 bit. Data were extracted using Feature Extraction software 10.7 (Agilent Technologies), and raw data were normalized using the Quantile algorithm and limma packages in R.

### 2.6 Reverse transcription and qPCR

A total of 200 ng of purified RNA was reverse transcribed to cDNA using a ReverTra Ace qPCR RT kit (FSQ-101, TOYOBO Co., Ltd., Life Science Department, Osaka, Japan) according to the protocols recommended by the manufacturer. Two microliters of cDNA was mixed with 10 μL of PowerUp™ SYBR™ Green Master Mix (A25742, Thermo Fisher Scientific, Waltham, MA, United States) and 2 μL of primers mix. qPCR was performed on a QuantStudio 7 Flex Real-time PCR System (Thermo Fisher Scientific) as follows: 50°C for 2 min, 95°C for 10 min, followed by 40 cycles of 95°C for 15 s and 60°C for 1 min, following the melting curve stage. The expression threshold for each lncRNA detector was automatically determined.

We calculated 2^(−ΔCT)^ and used this statistic to determine relative gene expression values. The relative amount of lncRNA in PBMCs was normalized against GAPDH. The primer sequences for qPCR used in this study are shown in Supplementary Table 1.

### 2.7 WGCNA

The WGCNA package (R 4.2.1) was used to construct a gene co-expression network and screen crucial genes significantly associated with TB. Among the expression profiles, the top 25% of genes with higher median expression values were used as the input (Chen et al., 2022). In the present study, we firstly used the function pickSoftThreshold and chose a soft-threshold *R^2^* value of 0.85. An adjacency matrix was performed into a topological overlap matrix (TOM) as well as the corresponding dissimilarity. Then, a hierarchical clustering tree diagram of the corresponding dissimilarity matrix was constructed to classify similar gene expression into different gene coexpression modules. Moreover, module-trait associations between modules and clinical feature information were calculated to selected the optimum module. Then, we estimated the gene significance (GS) value for each gene’s traits and module membership (MM) in the hub module. Finally, genes in the module were screened as potential TB-related genes based on a GS value > 0.90 and MM value > 0.85 as thresholds (Ling et al., 2023).

### 2.8 Enrichment analysis

Gene ontology (GO) and Kyoto Encyclopedia of Genes and Genomes (KEGG) analyses were performed using the Database for Annotation Visualization and Integrated Discovery^1^(Sherman et al., 2022). Figures and graphics to display the resulting data were generated using tools from the website^2^ (Tang et al., 2023).

### 2.9 Statistical analysis

More details regarding statistical methods for transcriptome data processing and module establishment are covered in the above sections. Demographic information and qPCR data were calculated using SPSS software (v.4.0.1). Parametric data are expressed as the mean ± standard deviation, and differences were assessed using the Student’s *t*-test. Non-parametric data are expressed as median (range), and differences were assessed using the Mann–Whitney *U*-test. Receiver operating characteristic (ROC) curves were constructed to determine the area under the curve (AUC) and evaluate the diagnostic value of biomarkers. Principal component analysis was performed in Python (v3.9.6). The regulation network was generated using Cytoscape (v3.10.1). Differences were considered statistically significant at *p* < 0.05.

## 3 Results

### 3.1 Characteristics of the study population

**Figure 1:**
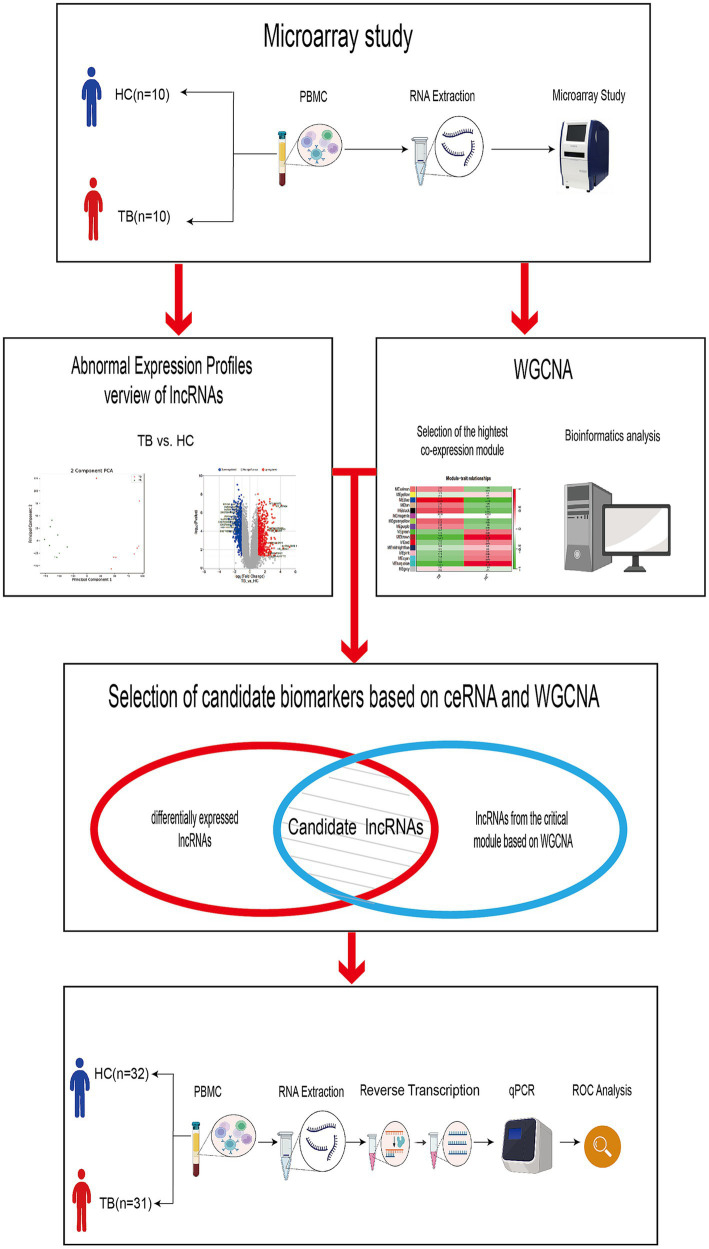
Work flow of the study. The discovery set included 8 TB patients and 9 HCs. The validation set included 31 TB patients and 32 HCs.

A total of 10 TB patients and 10 age- and gender-matched HCs who satisfied the inclusion and exclusion criteria were included in the discovery set for high-throughput ceRNA microarray analysis. Three of the samples (2 TB and 1 HC) were excluded from final analysis because of inferior raw microarray data quality. In addition, another 31 TB patients and 32 HCs were enrolled in the validation set for candidate biomarker validation and diagnostic performance analysis. Demographic information regarding the study population is summarized in Table 1. The work flow of this study is shown in Figure 1 (Created with BioRender.com).

**Table 1:** Demographic characteristics of the study population.

Study complex	Variables	TB	HCs	*p*-value
Discovery set	n^a^	8	9	
	Age (mean ± SD^b^)	59 ± 18	57 ± 12	0.790
	Male/female	6/2	4/5	0.335
Validation set	n	31	32	
	Age (mean ± SD)	57 ± 19	52 ± 17	0.300
	Male/female	26/5	16/16	0.007

^a^n, number of subjects. ^b^SD, standard deviation.

### 3.2 Overview of differential lncRNA expression profiles

**Figure 2:**
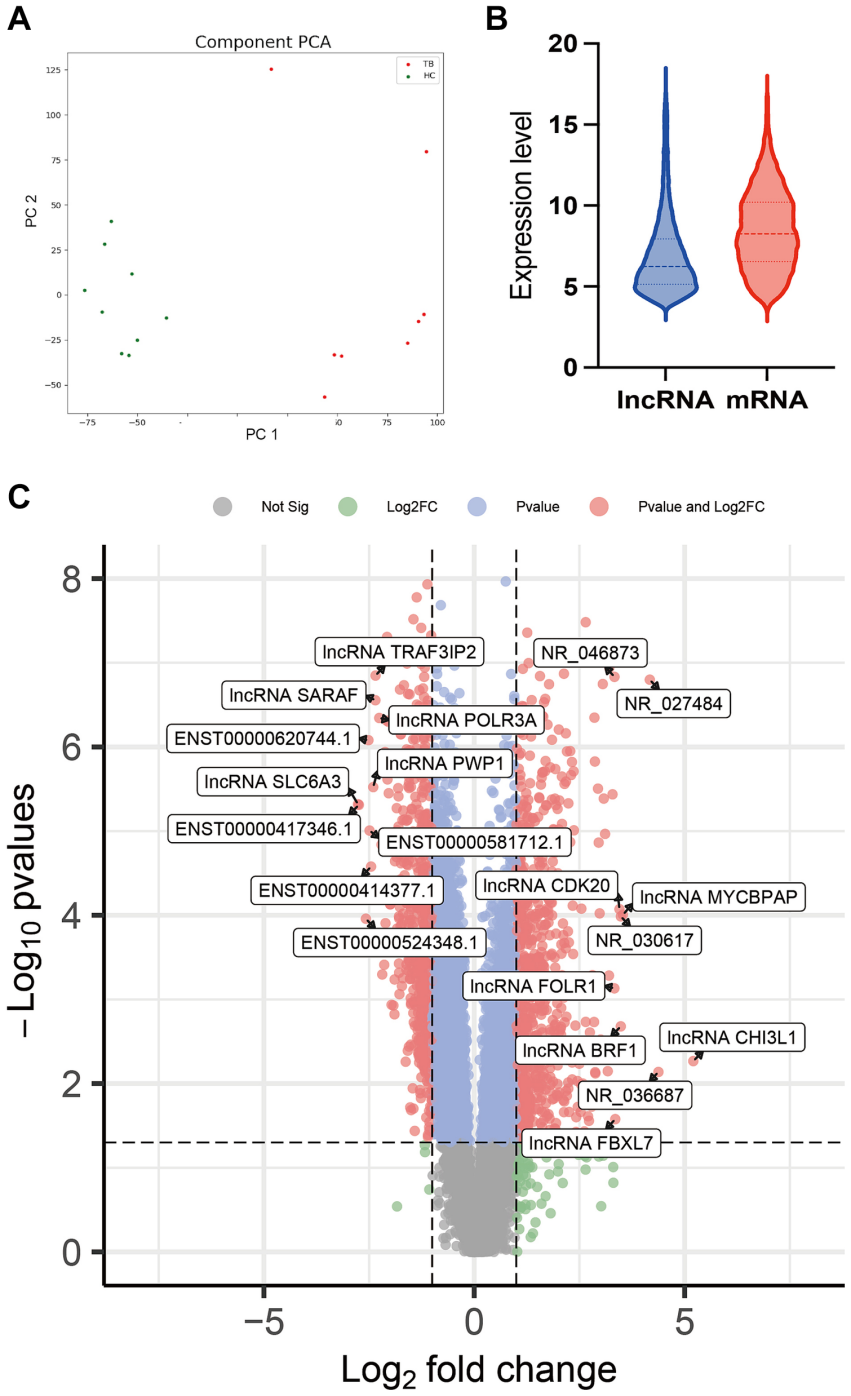
Comparison of lncRNA expression data between TB patients and HCs. **(A)** Principal component analysis of lncRNA expression profile in TB patients and HCs. **(B)** Volcano plot of the differentially expressed lncRNAs. **(C)** Expression patterns of lncRNAs and mRNAs in PBMCs.

Raw microarray data from 17 samples, including samples from 8 TB patients and 9 HCs, were finally normalized. The cluster of 17 samples based on lncRNA expression levels is shown by principal component analysis in a two-dimensional coordinate system (Figure 2A). A total of 1,372 lncRNAs differentially expressed between the TB patients and HCs were identified (fold-change >2 or <0.5 and *p* < 0.05), including 738 upregulated lncRNAs and 634 downregulated lncRNAs in the TB group (Figure 2B). The top 20 differentially expressed lncRNAs, including 10 upregulated and 10 downregulated, are listed in Supplementary Table 2. Furthermore, the results showed that average expression levels of lncRNAs in human PBMCs were lower than those of mRNAs (Figure 2C), in agreement with previous research (Mo et al., 2019).

### 3.3 Identification of key modules by WGCNA and enrichment analysis

**Figure 3:**
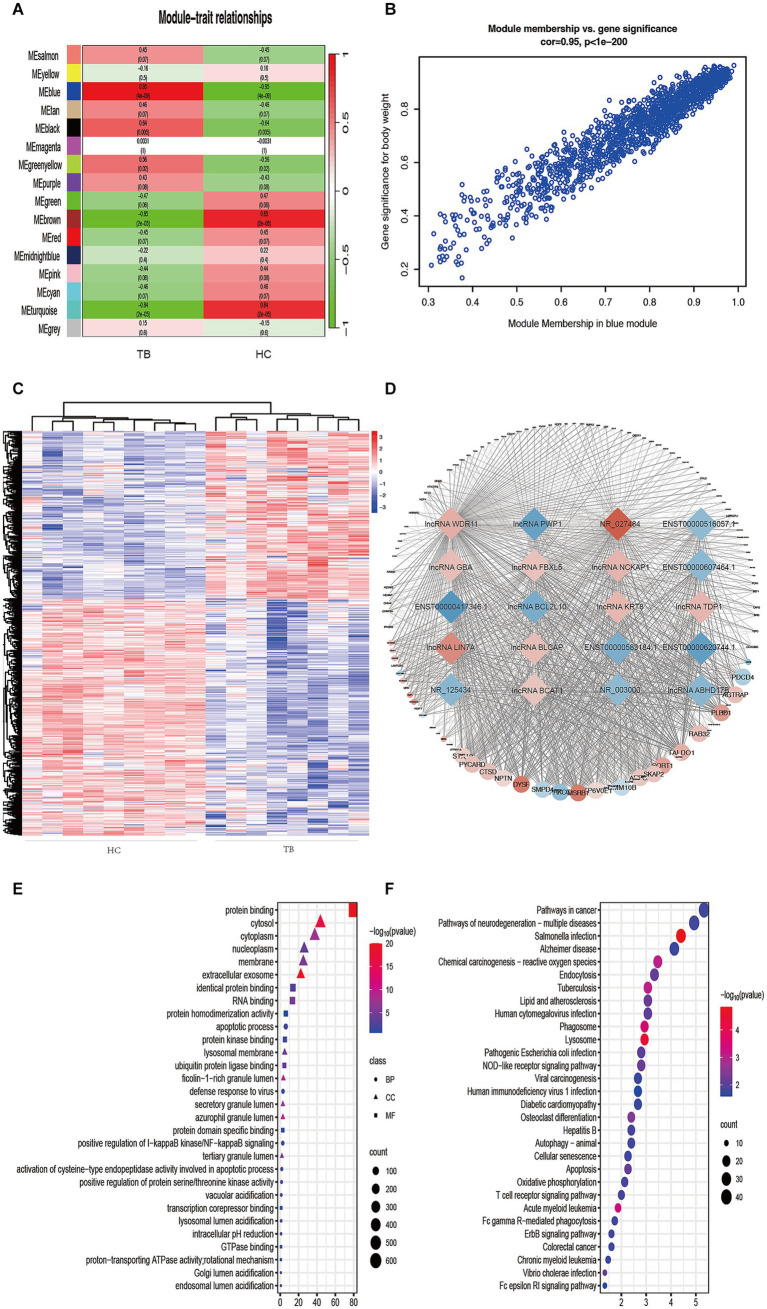
Identification of TB-related modules and key genes. **(A)** Analysis of correlations between the modules and TB; *p*-values are shown. **(B)** Scatter plot analysis of the blue module. Key genes were screened out in the upper-right area where GS > 0.90 and MM > 0.85. **(C)** Cluster analysis of the differentially expressed lncRNAs in the blue module. The samples were successfully clustered into 2 groups based on the lncRNA profile, and each group matched exactly to the clinical groupings of the TB patients and HCs. **(D)** Co-expression networks of selected genes in the blue module. Diamonds indicate lncRNAs. Circles indicate mRNAs. Red indicates upregulation. Blue indicates downregulation. **(E)** GO enrichment analysis. BP, biological process; MF, molecular function; CC, cellular component. **(F)** KEGG enrichment analysis. Colors indicate the *p*-value for each term.

To further understand the gene expression patterns in TB patients, a gene co-expression network was built using WGCNA (Supplementary Figure S1A). The top 25% of genes with higher median expression values were incorporated into the WGCNA (including 3,729 lncRNAs and 2,824 mRNAs). A power of β = 8 (*R^2^* = 0.85) was selected as the soft-thresholding parameter for scale-free network construction (Supplementary Figure S1B). Next, an adjacency matrix and topological overlap matrix were constructed. All genes were divided into different modules, and each module was assigned a different color. Sixteen modules were identified based on average hierarchical clustering and dynamic tree clipping (Supplementary Figure S1C). The correlation between each module and TB was assessed based on module-trait relationship. The results of the module-trait relationship analyses were shown in Figure 3A and indicated that the blue module had the highest correlation with TB (*r* = 0.95, *p* = 4 × 10^−9^), which indicated the genes in the blue module were highly associated with TB. The blue module was therefore selected as the meaningful module for further analysis.

In the blue module, correlations between MM and GS (Cor = 0.95) were observed by scatter plot analysis and cluster analysis (Figures 3B,C). Finally, lncRNAs in the blue module were screened as potential TB-related genes based on the criteria MM > 0.85, GS > 0.90, and *p* < 0.05 as thresholds. All of the selected lncRNAs also showed significantly different expression levels (*p* < 0.05 and fold-change >2 or < 0.5) between the TB and HC groups in the microarray results. Accordingly, we screened the top 10 significantly upregulated and 10 significantly downregulated genes for further validation, as shown in Table 2 and Figure 3D.

**Table 2:** The top 20 differentially expressed lncRNAs identified in blue module.

Genes	*p*-values	FC^a^(TB^b^/HC^c^)	Chromosome	Start	End	Relation	Associated_gene	Associated_gene_description
ENST00000417346.1	4.88E-06	0.149	chr20	57,384,160	57,393,062	antisense	RAE1	Ribonucleic acid export 1 [Source:HGNC Symbol;Acc:HGNC:9828]
ENST00000620744.1	8.32E-07	0.175	chr18	32,031,035	32,031,359	sense_intronic	RNF125	ring finger protein 125, E3 ubiquitin protein ligase [Source:HGNC Symbol;Acc:HGNC:21150]
lncRNA PWP1	3.00E-06	0.189	chr12	107,333,942	107,335,261	sense_intronic_ncRNA	BTBD11	BTB (POZ) domain containing 11 [Source:HGNC Symbol;Acc:HGNC:23844]
lncRNA BCL2L10	1.15E-06	0.230	chr15	52,123,558	52,128,273	sense_intronic_ncRNA	GNB5	Guanine nucleotide binding protein (G protein), beta 5 [Source:HGNC Symbol;Acc:HGNC:4401]
ENST00000583184.1	6.96E-08	0.236	chr18	32,018,829	32,111,779	processed_transcript	RNF125	Ring finger protein 125, E3 ubiquitin protein ligase [Source:HGNC Symbol;Acc:HGNC:21150]
NR_125434	9.83E-06	0.281	NT_187607.1	560,536	575,265	bidirectional	-	-
lncRNA ABHD17B	3.09E-06	0.286	chr9	71,750,000	71,768,513	sense_intronic_ncRNA	TMEM2	Transmembrane protein 2 [Source:HGNC Symbol;Acc:HGNC:11869]
ENST00000607464.1	4.14E-07	0.293	chr3	16,314,439	16,314,987	antisense	OXNAD1	Oxidoreductase NAD-binding domain containing 1 [Source:HGNC Symbol;Acc:HGNC:25128]
NR_003000	7.07E-07	0.298	chr17	7,906,122	7,906,260	intronic_sense	CHD3	Chromodomain helicase DNA binding protein 3 [Source:HGNC Symbol;Acc:HGNC:1918]
ENST00000516057.1	7.05E-07	0.299	chr1	15,542,165	15,542,304	scaRNA	DNAJC16	DnaJ heat shock protein family (Hsp40) member C16 [Source:HGNC Symbol;Acc:HGNC:29157]
lncRNA BCAT1	7.51E-07	2.547	chr12	24,967,126	24,967,504	lincRNA	-	-
lncRNA FBXL5	5.37E-07	2.660	chr4	15,492,728	15,492,972	antisense_lncRNA	CC2D2A	Coiled-coil and C2 domain containing 2A [Source:HGNC Symbol;Acc:HGNC:29253]
lncRNA GBA	6.78E-06	2.865	chr1	155,231,271	155,233,637	lincRNA	GBA	Glucosidase, beta, acid [Source:HGNC Symbol;Acc:HGNC:4177]
lncRNA BLCAP	1.18E-05	2.931	chr20	37,602,851	37,603,185	lincRNA	-	-
lncRNA KRT8	2.69E-07	3.220	chr12	52,949,215	52,949,954	sense_intronic_ncRNA	KRT18	Keratin 18, type I [Source:HGNC Symbol;Acc:HGNC:6430]
lncRNA TDP1	4.93E-06	3.260	chr14	90,107,674	90,107,995	sense_intronic_ncRNA	KCNK13	potassium channel, two pore domain subfamily K, member 13 [Source:HGNC Symbol;Acc:HGNC:6275]
lncRNA NCKAP1	1.80E-07	3.427	chr2	184,596,216	184,599,048	antisense_lncRNA	ZNF804A	Zinc finger protein 804A [Source:HGNC Symbol;Acc:HGNC:21711]
lncRNA WDR11	1.83E-06	3.803	chr10	120,843,216	120,844,603	lincRNA	WDR11	WD repeat domain 11 [Source:HGNC Symbol;Acc:HGNC:13831]
lncRNA LIN7A	1.49E-06	7.294	chr12	80,792,519	80,795,906	sense_intronic_ncRNA	LIN7A	Lin-7 homolog A (*C. elegans*) [Source:HGNC Symbol;Acc:HGNC:17787]
NR_027484	1.59E-07	18.012	chr1	143,874,742	143,883,733	exonic_sense	HIST2H2BB	Histone cluster 2, H2bb (pseudogene) [Source:HGNC Symbol;Acc:HGNC:20654]

^a^FC, Fold change; ^b^TB, Tuberculosis; ^c^HC, Health control.

GO and KEGG analyses were performed in order to predict the biological function of the critical module. The results of GO enrichment analysis are shown in Figure 3E. In the Biological Process category, most genes were enriched in regulation of apoptotic processes. In the Cellular Component category, most genes were enriched in the cytosol. In the Molecular Function category, most genes were enriched in protein binding. KEGG pathway analysis indicated that the genes in the blue module were enriched in many pathways, including *Salmonella* infection, lysosome, phagosome, acute myeloid leukemia, TB, and chemical carcinogenesis–reactive (Figure 3F). The result of GO and KEGG analyses showed that most genes in blue module enriched in immune-related biological process and pathway, such as apoptosis, autophagy and so on. The above results suggest that genes in blue module may play an important role in host immunity against tuberculosis infection.

### 3.4 Verification of lncRNAs by qPCR in the discovery and validation sets

**Figure 4:**
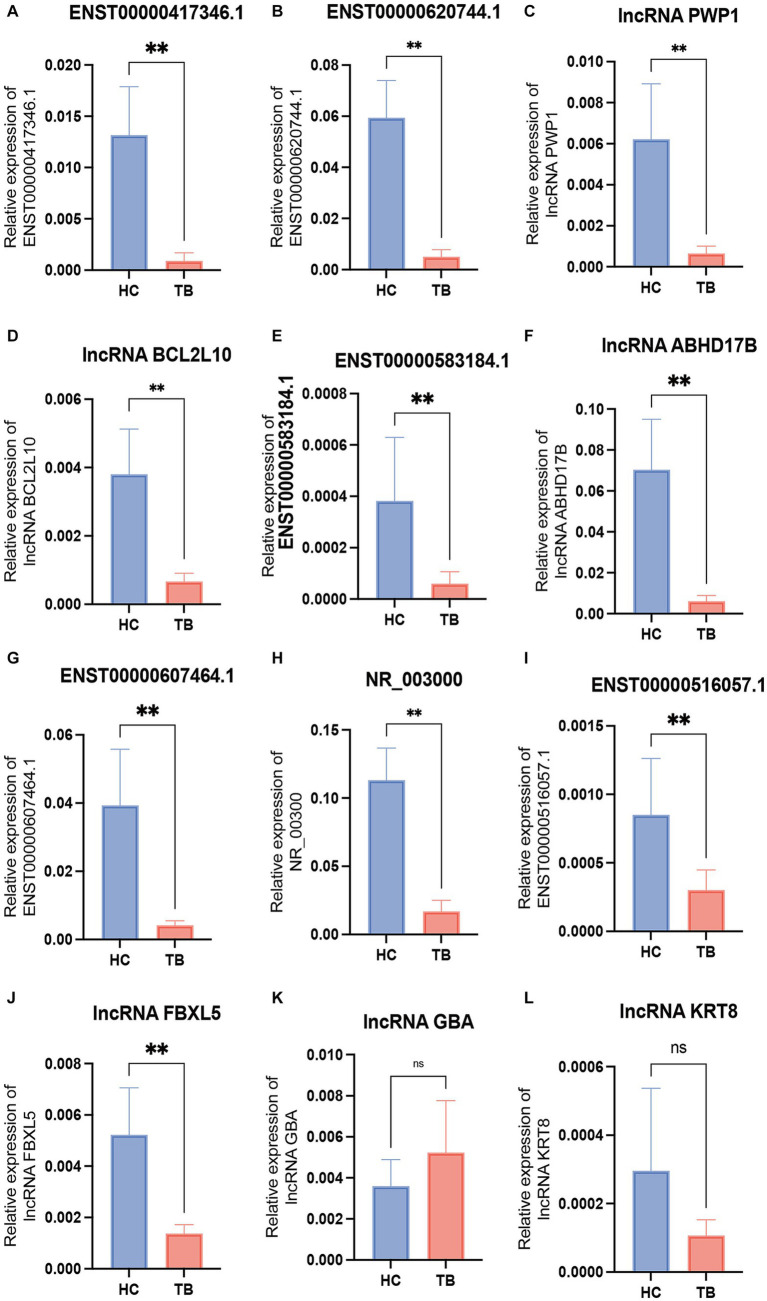
Validation of the differentially expressed lncRNAs by qPCR in the discovery set. The 12 differential lncRNAs were validated by qPCR in the discovery set. Ten of these lncRNAs showed the same expression pattern as in the microarray analysis **(A–L)**. NS, not significant; ^**^p < 0.01. Data presented as mean ± standard deviation.

Among the top 10 upregulated and top 10 downregulated lncRNAs in the blue module that were associated with TB, 12 lncRNAs were selected for further validation in the discovery set. The other 8 lncRNAs were not validated due to the highly conserved sequence relative to the encoding gene or a lack of specific primers for validation. Among the 12 differentially expressed genes, there were 3 upregulated lncRNAs (lncRNA GBA, lncRNA FBXL5 and lncRNA KRT8) and 9 downregulated lncRNAs (lncRNA periodic tryptophan protein 1 [PWP1], ENST00000620744.1, NR_003000, ENST00000417346.1, lncRNA BCL2L10, ENST00000516057.1, lncRNA ABHD17B, ENST00000607464.1 and ENST00000583184.1) in the TB group in the microarray analysis. qPCR analysis showed that the expression levels of 9 lncRNAs differed significantly and were consistent with the microarray results, whereas the expression patterns of lncRNA FBXL5 and lncRNA KRT8 were not consistent with the microarray results; there was no significant difference in the expression levels of lncRNA GBA between the TB and HC groups (Figure 4; Table 3).

**Table 3:** Differentially expressed lncRNAs identified by qPCR.

Gene	Discovery sample set		Validation sample set	
lncRNAs	*P*-value	FC^a^(TB^b^/HC^c^)	*P*-value	FC(TB/HC)
lncRNA ABHD17B	0.000	0.086	<0.0001	0.092
ENST00000516057.1	0.030	0.354	<0.0001	0.289
ENST00000583184.1	0.030	0.156	<0.0001	0.386
ENST00000620744.1	0.004	0.085	<0.0001	0.071
lncRNA KRT8	0.116	0.361	-	-
NR_003000	0.000	0.150	0.037	1.864
lncRNA PWP1	0.002	0.104	<0.0001	0.162
ENST00000607464.1	0.000	0.246	<0.0001	0.060
LncRNA GBA	0.159	1.420	-	-
LncRNA BCL2L10	0.001	0.463	<0.0001	0.533
ENST00000417346.1	0.001	0.181	<0.0001	0.166
LncRNA FBXL5	0.033	0.442	-	-

^a^FC, Fold change; ^b^TB, Tuberculosis; ^c^HC, Health control.

**Figure 5:**
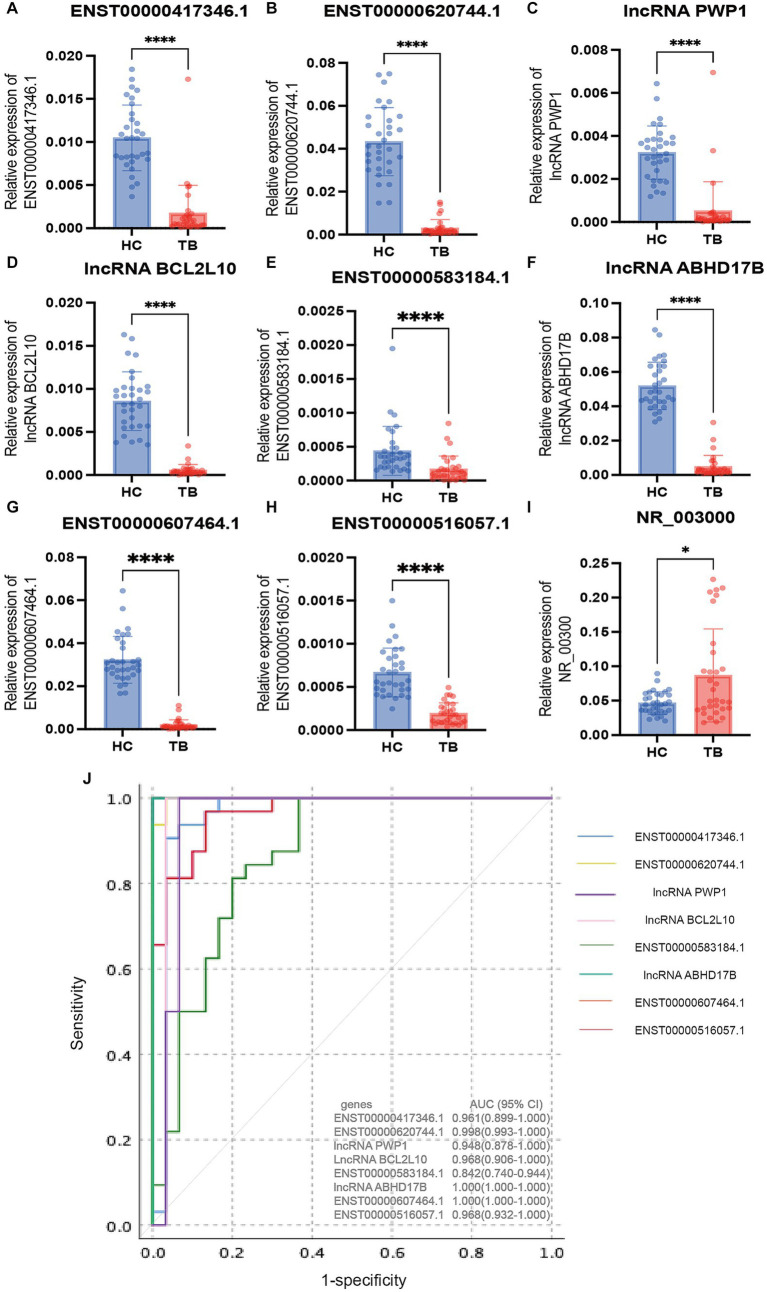
Validation of the differentially expressed lncRNAs by qPCR in the validation set. The 9 differential lncRNAs were validated by qPCR in the validation set **(A–I)**. Eight of these lncRNAs showed the same expression pattern as in the microarray analysis. The expression levels of lncRNA PWP1, ENST00000620744.1, ENST00000417346.1, lncRNA BCL2L10, ENST00000516057.1, lncRNA ABHD17B, ENST00000607464.1 and ENST00000583184.1 in the TB group were significantly lower than in the HC group, in which the expression patterns were consistent with the microarray results. The expression pattern of lncRNA NR_003000 was not consistent with the microarray results. The AUC values of the 8 lncRNAs are shown in **(J)**. ^*^p < 0.05; ^****^p < 0.0001. Data presented as mean ± standard deviation.

The 9 lncRNAs were further validated by qPCR in the validation sample set (31 TB patients and 32 HCs). As shown in Figure 5 and Table 4, the expression levels of lncRNA PWP1, ENST00000620744.1, ENST00000417346.1, lncRNA BCL2L10, ENST00000516057.1, lncRNA ABHD17B, ENST00000607464.1 and ENST00000583184.1 were significantly lower in the TB group than that in HC group. Furthermore, the expression patterns of 8 lncRNAs were consistent with the microarray results, whereas the expression pattern of lncRNA NR_003000 was not consistent with the microarray results.

**Table 4:** The AUC, sensitivity and specificity of the 8 differentially expressed lncRNAs in validation sample set.

Genes	Validation sample set			
	Cut-off	Sensitivity^a^ (95% CI^b^)	Specificity	AUC(95% CI)
lncRNA ABHD17B	0.0307	1.000 (0.943–1.000)	1.000 (0.9431.000)	1.000 (1.000–1.000)
ENST00000516057.1	0.0004	0.969 (0.892–0.991)	0.871 (0.769–0.934)	0.968 (0.932–1.000)
ENST00000583184.1	0.0002	0.844 (0.732–0.912)	0.742 (0.627–0.837)	0.842 (0.740–0.944)
ENST00000620744.1	0.0144	1.000 (0.943–1.000)	0.968 (0.892–0.991)	0.998 (0.993–1.000)
lncRNA PWP1	0.0008	1.000 (0.943–1.000)	0.903 (0.807–0.956)	0.948 (0.878–1.000)
ENST00000607464.1	0.0138	1.000 (0.943–1.000)	1.000 (0.943–1.000)	1.000 (1.000–1.000)
LncRNA BCL2L10	0.0034	1.000 (0.943–1.000)	0.968 (0.892–0.991)	0.968 (0.906–1.000)
ENST00000417346.1	0.0050	0.938 (0.848–0.975)	0.935 (0.848–0.975)	0.961 (0.899–1.000)

^a^AUC, Area under curve; ^b^CI, Confidence intervals.

### 3.5 Diagnostic performance of the differentially expressed lncRNAs

To evaluate the diagnostic accuracy of the 8 lncRNAs, an ROC curve was generated to determine the AUC, sensitivity, and specificity of each lncRNA in discriminating TB patients from HCs in the discovery and validation sets. As shown in Table 4 and Figure 5J, the ROC curve for the validation set showed that lncRNA ABHD17B (AUC = 1.000) and ENST00000607464.1 (AUC = 1.000) were the best lncRNAs in distinguishing the TB and HC groups, followed by ENST00000620744.1 (AUC = 0.998) and the lncRNA BCL2L10 (AUC = 0.967). As each lncRNA showed excellent diagnostic performance in differentiating TB patients and HCs, we did not analyze whether combining these differentially expressed lncRNAs would provide better diagnostic accuracy.

## 4 Discussion

TB remains a serious public health problem, particularly in China, due to the large number of TB patients, which generates a great burden and risk of transmission. Furthermore, common methods to diagnose TB depend on clinical, immunological, microscopic, radiographic, and bacterial culture (Acharya et al., 2020). However, due to equipment, technology, and sensitivity limitations, the common methods can not satisfy the requirements for TB diagnosis in clinical practice. Even the utility of the molecular diagnostic techniques, including GeneXpert/MTB, also have limitations in clinical application, since the utility rate of these molecular diagnostic tests was limited (47%) in people newly diagnosed with TB (World Health Organization, 2023). Therefore, novel and rapid diagnosis methods, including the host biomarkers-based assay, which present higher analytical sensitivity and reduce assay times, remain to be explored. One type of target molecule is lncRNAs, which act through a plethora of different mechanisms and interactors and function as important regulators in many aspects of biology. lncRNAs play important roles in a variety of biological processes, including development and immune responses (Jonas and Izaurralde, 2015; Marchese et al., 2017; McDonel and Guttman, 2019). A broader and more in-depth understanding of the regulatory mechanisms of host lncRNAs could contribute to the identification of novel targets for TB diagnosis or development of host-directed anti-TB therapies.

Characterizing the lncRNA-mRNA interaction patterns and connection between gene modules and TB could provide criteria for identifying functional lncRNA-mRNA relationships. However, the lncRNA-mRNA correlation patterns are far from clear. With the development of bioinformatics techniques, all types of expression profile data, such as transcriptome and single-cell sequencing data, can be re-analyzed from different dimensions. The identification of differentially expressed genes is the most classical and fundamental analyses and commonly used in screening novel biomarkers via a series of statistical algorithms to identify differentially expressed genes between subgroups. WGCNA is a topological network analysis approach that can establish the linkage between gene modules and clinical traits; genes classified into the same module are all linked to the selected clinical traits, which can then be used for subsequent analysis and experiments. Because it can be linked with clinical information, immunological state, biological function, and other specific characteristics, WGCNA can be used to efficiently screen biomarkers. As such, WGCNA has been used in numerous studies to identify biomarkers associated with other diseases. For example, Wen et al. used WGCNA to preliminarily screen protein biomarkers, and the results were then combined with enzyme-linked immunosorbent assay results to verify CCL19, C1Qb, CCL5, and HLA-DMB as potentially effective biomarkers for TB diagnosis (Wen et al., 2022). A study on pediatric sepsis verified that 4 lncRNAs (GSEC, NONHSAT160878.1, XR_926068.1, and RARA-AS1) identified by WGCNA were linked to prognosis based on function (Zhang et al., 2021). A large number of studies based on WGCNA have suggested that the unique algorithm tends to cause the expression network to be distributed, which is of paramount importance in the screening of biomarkers.

In the present study, we analyzed the expression profiles of lncRNAs in PBMCs from TB patients and HCs using a ceRNA microarray. A total of 1,372 differentially expressed genes were identified in TB patients, suggesting that the gene expression regulation network of lncRNAs is altered in individuals with TB. A subsequent WGCNA further identified the critical module and specific biomarkers. In addition, KEGG analysis showed that the blue module was significantly enriched in infection and immunity-related processes, including autophagy and apoptosis. Some lncRNAs in the blue module in our study, have been previously confirmed to participate in apoptosis by experiments, including the promotion of apoptosis by lncRNA PAXIP1 (Ma and Zheng, 2021) and lncRNA SLC9A3 (Li et al., 2021), while the inhibition of apoptosis by lncRNA EZR-AS1 (Yu et al., 2023). Meanwhile, recent studies have also shown that lncRNA EGOT (Liu et al., 2020) can inhibit autophagy, either by ceRNA interactive patterns or by posttranscriptional regulation of the ATG7/16 L1 (Wang I. K. et al., 2020). Autophagy and apoptosis are common kinds of programmed cell death to regulate inflammation and injury which played significant roles in anti-TB immune response (Liu et al., 2018). Increasing evidence suggests that not only mRNA, but also ncRNAs, participate in autophagy and apoptosis in TB occurrence and development (Wang Y. et al., 2020). For instance, it was proved that the lncRNA MIAT could regulate autophagy and apoptosis in macrophages infected by BCG through the miR-665/ULK1 signaling axis (Jiang et al., 2021). Furthermore, PCED1B-AS1, as an endogenous sponge, was involved in TNF-α-induced apoptosis and autophagy by targeting the miR-155/FOXO3 (Rheb) axis (Li et al., 2019). Therefore, these results indicated it was the genes in the blue module that associated with host anti-TB immune response, which were promising potential biomarkers and targets for TB diagnosis and treatment. In addition, the result of the Molecular Function category in GO analyses showed genes in blue module were most enriched in protein binding. As we known, the important function of the lncRNA was binding with RNA-binding proteins to regulate gene expression. For example, lncRNA EST12 suppresses antimycobacterial innate immunity through interaction with FUBP3 in *M.tb* infection (Yao et al., 2022). Therefore, these results also confirmed the reliability of our analysis.

Ultimately, 8 of the lncRNAs were selected to validate by qPCR, which exhibited superior diagnostic performance in the validation sample set, especially 2 of the 8 lncRNAs showed an AUC value of 1 in discriminating TB patients from HCs. Nevertheless, we also screened the differentially expressed lncRNAs using differential gene analysis based on fold-change, and also detected another group of top 10 up-regulated lncRNAs and top 10 down-regulated lncRNAs. Two of them (lncRNA MYCBPAP and lncRNA CHI3L1) were validated by qPCR and the diagnostic performance of these two lncRNAs were decreased (AUC = 0.915 and AUC = 0.656), respectively, indicating less ability to discriminate TB patients from HCs. These results suggest that WGCNA is a more beneficial tool for biomarker screening, than the traditional differential gene analysis.

The present study confirmed that lncRNAs aberrantly expressed in PBMCs of TB patients are potentially useful biomarkers for diagnosis of TB and also appear to be associated with regulating the host immune response to TB infection. Some research has identified critical lncRNA and further focused on the role of lncRNAs in the immune regulation of *M.tb* infection. For example, lncRNA EST12, which is found mainly in the cytoplasm, interacted with the transcription factor far upstream element-binding protein 3 (FUEBP3) to suppresses the NLRP3 inflammasome assembly and gasdermin D–mediated pyroptosis–IL-1β immune pathway (Yao et al., 2022). Furthermore, in CD8^+^ T cells, CD244 signaling drives lncRNA-CD244 expression which was selected based on microarray and lncRNA-CD244 inhibits IFN-γ/TNF-α expression by mediates H3K27 trimethylation at infg/tnfa loci (Wang et al., 2015). Therefore, further in-depth analyses of the functions and regulatory mechanisms of the crucial lncRNAs in the blue module that were screened in this study may provide clues to elucidate the pathogenesis of TB occurrence and to develop new TB treatment strategy.

Eight differentially expressed lncRNAs identified in our study have not been reported elsewhere to date in TB field, although there was a research on lncRNA WDR11 divergent transcript (lncRNA WDR11-AS1) suggested that the lncRNA WDR11-AS1 had an effect on inflammation (Huang et al., 2023). In contrast to miRNAs and mRNA, there are no standard rules for naming lncRNAs, and the most commonly used naming methods are primarily based on the function or origin of the encoding gene. For example, lncRNA BC050410, which is derived by CD244 signaling in CD8^+^ T cells, is located nearby the 5′ UTR of Glutathione S-transferase T 1 (GSTT1), so it is named as lncRNA AS-GSTT1 due to its genomic context, and also can be termed as lncRNA-CD244 that is associated with its function (Wang et al., 2015). Although the varied methods of naming brought inconvenience for research on lncRNA, there was no doubt that lncRNAs played an important role in TB related immune response and researches on lncRNAs needs to be further refined and enriched (Yan et al., 2018).

There are some limitations to our study. First, the sample size in the discovery set for the microarray analysis was moderate. Although we enrolled an independent sample set to validate the differentially expressed lncRNAs, we cannot rule out the potential for bias resulting from sample heterogeneity. Second, this microarray analysis was performed in 2019. Although we ultimately identified 8 candidate biomarkers, the molecular characteristics of the lncRNAs need to be verified by in-depth experiments, and as the database iterates, the types and quantities of lncRNAs may update, which could result in an alteration of the TB-specific lncRNA profile, more or less. However, the major types of RNAs identified in our study are very similar to those reported in previous studies, which confirms the accuracy of our microarray results (Mo et al., 2019).

In conclusion, our study characterized the lncRNA profiles in PBMCs of TB patients, resulting in the identification of a critical module associated with TB. Furthermore, a total of 8 lncRNAs differentially expressed between the TB and HC groups were identified and were shown as promising biomarkers for discriminating TB from HCs.

## Data availability statement

The datasets presented in this study can be found in online repositories. The names of the repository/repositories and accession number (s) can be found at: https://www.ncbi.nlm.nih.gov/, GSE249824.

## Ethics statement

The studies involving humans were approved by Ethics Committee of the Beijing Chest Hospital, Capital Medical University. The studies were conducted in accordance with the local legislation and institutional requirements. The participants provided their written informed consent to participate in this study.

## Author contributions

JD: Data curation, Writing – original draft. RS: Validation, Writing – original draft. XS: Validation, Writing – original draft. YW: Validation, Writing – original draft. QL: Resources, Writing – original draft. ZhZ: Resources, Writing – original draft. HJ: Resources, Writing – original draft. MH: Resources, Writing – original draft. CZ: Supervision, Writing – original draft. QS: Resources, Writing – original draft. BD: Resources, Writing – original draft. AX: Resources, Writing – original draft. ZL: Supervision, Writing – original draft. LZ: Supervision, Writing – original draft. LP: Project administration, Supervision, Writing – review & editing. ZoZ: Project administration, Supervision, Writing – review & editing.

## Footnotes

^1^https://david.ncifcrf.gov/home.jsp/

^2^www.bioinformatics.com.cn

## Glossary

ATB: Active tuberculosis
AUC: Area under curve
DUSP3: Dual specificity phosphatase 3
GSTT1: Glutathione S-transferase T 1
GO: Gene Ontology
GS: Gene significance
GBP5: Guanylate-binding protein
HBV: Hepatitis B virus
HC: Health control
HCV: Hepatitis C virus
HIV: Human immunodeficiency virus
KEGG: Kyoto Encyclopedia of Genes and Genomes
KLF2: Krupple-like factor 2
lncRNAs: Long noncoding RNAs
lncRNA WDR11-AS1: lncRNA WDR11 divergent transcript
*M.tb*: *Mycobacterium tuberculosis*
MM: Module membership
ncRNA: Noncoding RNA
PBMC: Peripheral blood mononuclear cell
qPCR: Real-time quantitative polymerase chain reaction
ROC: Receiver operating characteristic
RT: Reverse transcription
TB: Tuberculosis
WGCNA: Weighted gene co-expression network analysis
