# Supplementary material for: Identification of important modules and biomarkers in tuberculosis based on WGCNA
Source: Front Microbiol. 2024 Feb 8;15:1354190. doi: 10.3389/fmicb.2024.1354190 (PMC10882270; doi:10.3389/fmicb.2024.1354190)
Supplement: Supplementary file 1 [file Data_Sheet_1.docx]

Supplementary Material

# Supplementary Figures and Tables

**Supplementary Table S1. The primer sequences for qPCR**

| genes | forward primer（5’ - 3’） | reverse primer（5’ - 3’） |
| --- | --- | --- |
| ENST00000583184.1 | GGAGACAGCAGCAAGGTGTGTA | ACATTTTCTACAGAAACACAGGCC |
| lncRNA ABHD17B | CCCAGAGAGAAAGTCAAAAGCA | TATACACACACTTACCATCTGGAGC |
| ENST00000620744.1 | TTGGGTTTCTGAGTTTGAGTCTG | AGAAATCGGAATCAGACACGG |
| NR_003000 | CTCAGCCGAGCCTAGAGTAGAG | CCAGGAGAGGCTCCTTGCTT |
| ENST00000516057.1 | CCTCAGCCAAGCCTACAGTAGA | CCAGGTGAGGCTCCTTACTCAA |
| lncRNA KRT8 | ATCCGCTTAACCCTCCAATAGT | TTTACAGAGGAAGTGGACAGCAT |
| lncRNA PWP1 | GAGAAGCATGAGTGAGCCAAGA | CCTACCCAACCTGGCTTACG |
| lncRNA GBA | TTCCATGTACTGCCGCTGC | TCTTCCTGCCCACCCAATC |
| ENST00000417346.1 | CTTGGGCCTTCCACACCAGT | GAGCCAGGGCATCCATTTG |
| lncRNA BCL2L10 | GAGCATCCAGCGTGGACTTCT | GCGCTAGTTTCATTCTGGTCCT |
| lncRNA FBXL5 | AGGTGGTGAAGCAGGCATCA | GTGGAAGAGTGGGTGTCACTGTTA |
| ENST00000607464.1 | GAATCAGCAGGGTGTAGAGAAGC | ATGTTGGCTGTATTTGCTATGG |

**Supplementary Table S 2. The top 20 differentially expressed lncRNAs identified by ceRNA-microarray.**

| Genes | p value | FC(TB/HC) | Chromosome | Start | End | Relation | Associated_gene | Associated_gene_description |
| --- | --- | --- | --- | --- | --- | --- | --- | --- |
| lncRNA SLC6A3 | 4.80E-06 | 0.147 | chr5 | 1383159 | 1386409 | lincRNA | SLC6A3 | solute carrier family 6 (neurotransmitter transporter), member 3 [Source:HGNC Symbol;Acc:HGNC:11049] |
| ENST00000417346.1 | 4.88E-06 | 0.149 | chr20 | 57384160 | 57393062 | antisense | RAE1 | ribonucleic acid export 1 [Source:HGNC Symbol;Acc:HGNC:9828] |
| ENST00000524348.1 | 0.000110 | 0.168 | chr8 | 122808770 | 122815774 | sense_intronic | ZHX2 | zinc fingers and homeoboxes 2 [Source:HGNC Symbol;Acc:HGNC:18513] |
| ENST00000620744.1 | 8.32E-07 | 0.175 | chr18 | 32031035 | 32031359 | sense_intronic | RNF125 | ring finger protein 125, E3 ubiquitin protein ligase [Source:HGNC Symbol;Acc:HGNC:21150] |
| ENST00000581712.1 | 9.86E-06 | 0.178 | chr18 | 738058 | 739662 | sense_intronic | YES1 | YES proto-oncogene 1, Src family tyrosine kinase [Source:HGNC Symbol;Acc:HGNC:12841] |
| ENST00000414377.1 | 2.64E-05 | 0.183 | chr1 | 184408337 | 184412360 | sense_intronic | C1orf21 | chromosome 1 open reading frame 21 [Source:HGNC Symbol;Acc:HGNC:15494] |
| lncRNA PWP1 | 3.00E-06 | 0.189 | chr12 | 107333942 | 107335261 | sense_intronic_ncRNA | BTBD11 | BTB (POZ) domain containing 11 [Source:HGNC Symbol;Acc:HGNC:23844] |
| lncRNA SARAF | 2.79E-07 | 0.196 | chr8 | 30094656 | 30095405 | antisense_lncRNA | LEPROTL1 | leptin receptor overlapping transcript-like 1 [Source:HGNC Symbol;Acc:HGNC:6555] |
| lncRNA TRAF3IP2 | 1.42E-07 | 0.197 | chr6 | 111767512 | 111767805 | sense_intronic_ncRNA | FYN | FYN proto-oncogene, Src family tyrosine kinase [Source:HGNC Symbol;Acc:HGNC:4037] |
| lncRNA POLR3A | 4.53E-07 | 0.209 | chr10 | 77816032 | 77820021 | sense_intronic_ncRNA | DLG5 | discs, large homolog 5 (Drosophila) [Source:HGNC Symbol;Acc:HGNC:2904] |
| lncRNA FOLR1 | 0.000739 | 10.047 | chr11 | 72172454 | 72177954 | lincRNA | RP11-807H22.6 | - |
| NR_046873 | 1.47E-07 | 10.065 | chr3 | 188941714 | 188947639 | intergenic | TPRG1 | tumor protein p63 regulated 1 [Source:HGNC Symbol;Acc:HGNC:24759] |
| lncRNA FBXL7 | 0.0264 | 10.197 | chr5 | 15451995 | 15452553 | lincRNA | - | - |
| lncRNA CDK20 | 8.47E-05 | 10.926 | chr9 | 88066438 | 88076674 | lincRNA | - | - |
| lncRNA BRF1 | 0.00209 | 11.173 | chr14 | 105644495 | 105648555 | lincRNA | MIR8071-2 | microRNA 8071-2 [Source:HGNC Symbol;Acc:HGNC:49958] |
| NR_030617 | 0.000103 | 11.176 | chr14 | 100875032 | 100875104 | exonic_sense | MIR136 | microRNA 136 [Source:HGNC Symbol;Acc:HGNC:31522] |
| lncRNA MYCBPAP | 9.85E-05 | 11.570 | chr17 | 50532737 | 50536346 | sense_intronic_ncRNA | EPN3 | epsin 3 [Source:HGNC Symbol;Acc:HGNC:18235] |
| NR_027484 | 1.59E-07 | 18.012 | chr1 | 143874742 | 143883733 | exonic_sense | HIST2H2BB | histone cluster 2, H2bb (pseudogene) [Source:HGNC Symbol;Acc:HGNC:20654] |
| NR_036687 | 0.00728 | 20.760 | NW_018654717.1 | 401414 | 403957 | exonic_sense | - | - |
| lncRNA CHI3L1 | 0.00541 | 36.881 | chr1 | 203212834 | 203218021 | sense_intronic_ncRNA | CHIT1 | chitinase 1 [Source:HGNC Symbol;Acc:HGNC:1936] |

1. FC: Fold change;
2. TB: Tuberculosis;
3. HC: Health control.

## Supplementary Figures


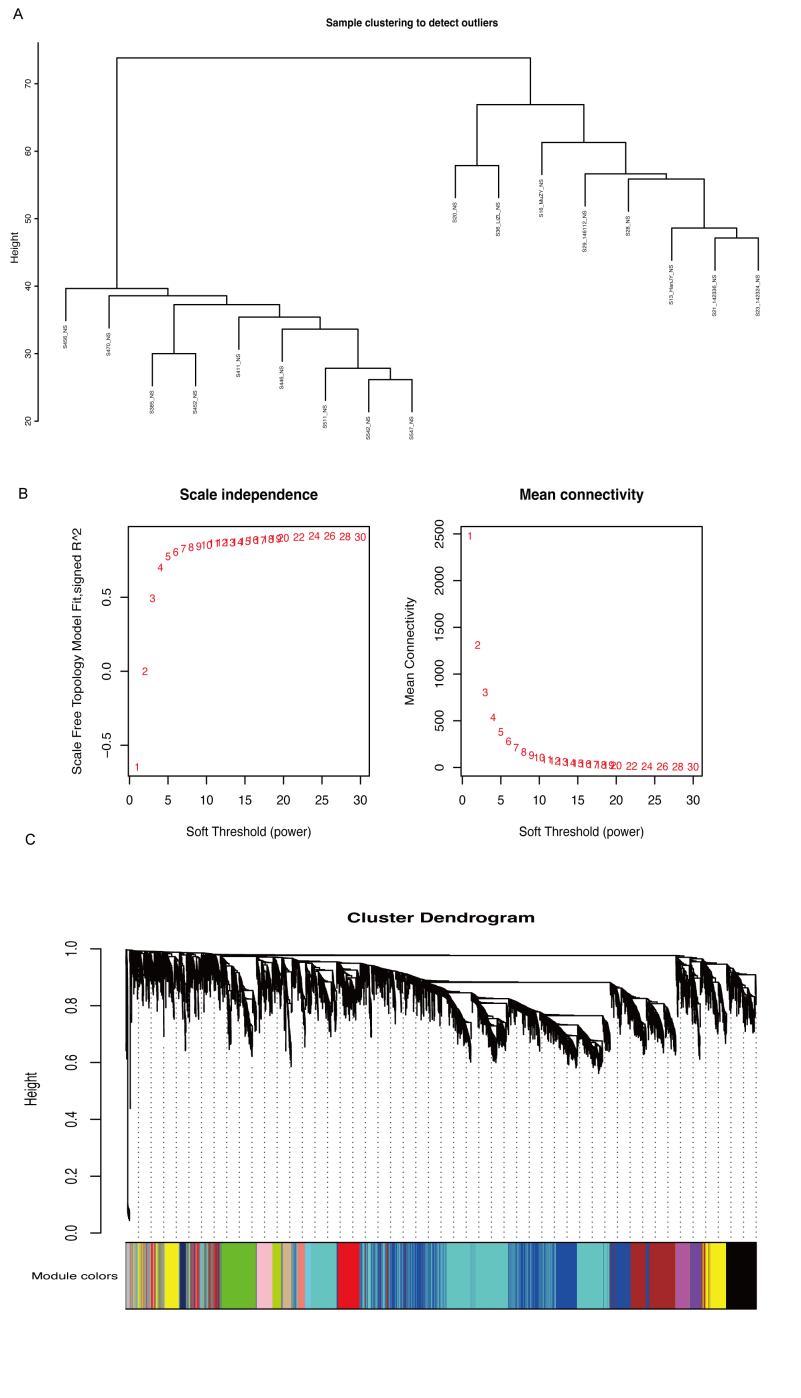


**Supplementary Figure 1**. Analysis of network topology for soft-thresholding powers and gene dendrogram for WGCNA. **(A, B)** Sample clustering tree and graphs of scale independence, mean connectivity, and scale-free topology; the appropriate soft-power was 8. **(C)** Cluster dendrogram of the co-expression network modules.
